# Pneumothorax detection and segmentation from chest X-ray radiographs using a patch-based fully convolutional encoder-decoder network

**DOI:** 10.3389/fradi.2024.1424065

**Published:** 2024-12-11

**Authors:** Jakov Ivan S. Dumbrique, Reynan B. Hernandez, Juan Miguel L. Cruz, Ryan M. Pagdanganan, Prospero C. Naval

**Affiliations:** ^1^ Computer Vision and Machine Intelligence Group, Department of Computer Science, University of the Philippines-Diliman, Quezon City, Philippines; ^2^ Department of Mathematics, Ateneo de Manila University, Quezon City, Philippines; ^3^ Ateneo School of Medicine and Public Health, Pasig, Philippines; ^4^ Department of Radiology, The Medical City, Pasig, Philippines

**Keywords:** pneumothorax, automatic image segmentation, deep learning, convolutional neural network, Vision Transformer, lung pathology detection, chest X-rays, diagnostic radiology

## Abstract

Pneumothorax, a life-threatening condition characterized by air accumulation in the pleural cavity, requires early and accurate detection for optimal patient outcomes. Chest X-ray radiographs are a common diagnostic tool due to their speed and affordability. However, detecting pneumothorax can be challenging for radiologists because the sole visual indicator is often a thin displaced pleural line. This research explores deep learning techniques to automate and improve the detection and segmentation of pneumothorax from chest X-ray radiographs. We propose a novel architecture that combines the advantages of fully convolutional neural networks (FCNNs) and Vision Transformers (ViTs) while using only convolutional modules to avoid the quadratic complexity of ViT’s self-attention mechanism. This architecture utilizes a patch-based encoder-decoder structure with skip connections to effectively combine high-level and low-level features. Compared to prior research and baseline FCNNs, our model demonstrates significantly higher accuracy in detection and segmentation while maintaining computational efficiency. This is evident on two datasets: (1) the SIIM-ACR Pneumothorax Segmentation dataset and (2) a novel dataset we curated from The Medical City, a private hospital in the Philippines. Ablation studies further reveal that using a mixed Tversky and Focal loss function significantly improves performance compared to using solely the Tversky loss. Our findings suggest our model has the potential to improve diagnostic accuracy and efficiency in pneumothorax detection, potentially aiding radiologists in clinical settings.

## 1 Introduction

Semantic segmentation is essential in modern medical image analysis since it fosters the identification of anatomical structures (1) and the diagnosis of various diseases (2). In the past decade since the rise of deep learning, fully convolutional neural networks (FCNNs), especially “U-shaped” encoder-decoder architectures (3, 4), have produced state-of-the-art results in a variety of medical semantic segmentation applications (5, 6), to the extent that they have become the de-facto standard in the field (7). In a conventional U-Net (8) architecture, the encoder captures the local and global context in an image using a stack of convolutional and pooling layers, and the decoder enables precise localization through transposed convolutions and upsampling. The superior performance of U-Net is primarily attributed to its overlap-tile segmentation strategy and its combination of features from the encoder with intermediary outputs from the decoder so that a successive convolutional layer can learn to assemble a more precise output based on this recovered spatial information.

While the standard U-Net architecture has been effective in segmentation tasks, its model performance is limited by its hard-coded receptive field size (9) and the number of hidden layers in its encoder and decoder. Because of this difficulty in extracting multi-scale information, the conventional U-Net is limited in localizing structures of varying non-standard shapes and on variable positions relative to other regions on the image (10). To address this drawback, various convolutional modules such as dilated convolutions (11, 12) have been proposed to capture contextual information from larger receptive field without increasing filter size. Moreover, augmenting convolutional layers with self-attention mechanisms (3, 4) has also shown to better encode long-range dependencies.

Recently, numerous studies show that transformers can surpass traditional convolutional neural networks in many generic vision tasks (13, 14). However, convolutional networks (ConvNets) are still preferred over transformers for dense prediction (e.g., semantic segmentation) on images with rigid structure such as frontal chest X-rays. First, the inherent translational equivariance of ConvNets is an important inductive bias for vision tasks on radiographs and other structured images with repeated markings located on different parts of the image. Second, as a consequence of Vision Transformers (ViTs) not inherently exhibiting any image-specific inductive bias, they require larger model architecture and larger dataset sizes to learn the desired equivariance property. ConvNets therefore can outperform transformers on tasks such as medical image segmentation where the dataset is costly to annotate and verify. Lastly, ViT’s self-attention design has a quadratic complexity with respect to the input size, making it not suitable for situations that require real-time inference under low-resource constraint (e.g., deployment in emergency rooms).

In this work, we test the limits of fully convolutional neural networks (FCCNs) in the task of pneumothorax detection and segmentation on chest X-rays. Pneumothorax is a condition where air accumulates in the pleural space around the lungs, causing the lung to collapse partially or fully (15). It is a common disease in medical practice that affects young healthy people with a significant recurrence rate (16). Accurate detection of pneumothorax on chest X-rays is not always easy in practice since the disease’s sole visual marking on a radiograph is a thin displaced pleural line (17). Because of its life-threatening condition (18) coupled with a shortage of radiologists in developing countries such as the Philippines (19), there is a need to detect pneumothorax in patients accurately and quickly. In this work, we are interested in automating the detection and segmentation of pneumothorax on digital chest radiographs.

X-ray is the choice of imaging modality for this study as it provides a quick and accurate assessment of pneumothorax, allowing for prompt and appropriate treatment (20). While computer tomography (CT) scan and ultrasonography have been found to have higher sensitivity in detecting pneumothorax, X-rays have been shown to have at-par or even higher specificity over the two imaging modalities (21–23). Moreover, X-rays are a reliable and readily-available tool in hospitals and are often the first modality used to make and rule out the diagnosis of pneumothorax and to help guide further management decisions (20).

For this study, we introduce a patch-based fully convolutional encoder-decoder network aptly named as Pneumothorax Detection and Segmentation on Chest X-rays (P-DeSeRay). We compare our work with prior art and other FCNNs. Aside from architectural changes, we also study how the combination of loss functions affects model performance. We evaluate the effectiveness of our proposed method on the SIIM-ACR Pneumothorax Segmentation dataset (24) and our own curated dataset from the Radiology Department of The Medical City (TMC), a private hospital in Pasig City, Philippines. P-DeSeRay achieves state-of-the-art results on both datasets.

Our work’s main contributions are as follows:

• We proposed a fully convolutional encoder-decoder network using convolution modules deemed equivalent as their counterparts in Vision Transformers (ViTs) to bypass the quadratic complexity of the self-attention mechanism in ViTs. Specifically, we constructed a novel architecture in which (1) a convolutional encoder directly uses the embedded 2D patches to effectively capture long-range dependencies; and (2) a skip-connected decoder combines the extracted representations at different resolutions and predicts the desired segmentation output.
• We trained our proposed model using a novel combination of segmentation losses. In particular, we have shown that training our model on an unweighted mixed loss combining Tversky and Focal losses resulted to superior segmentation and detection performance when compared to training our model using Tversky loss solely.
• We validated the effectiveness of our proposed model on a public dataset and a locally curated dataset. P-DeSeRay achieves state-of-the-art detection and segmentation performance on both datasets compared to other convolutional networks and to radiologists’ diagnostic performance level.

## 2 Background

### 2.1 Fully convolutional segmentation networks

The foundational U-Net (8) has led to a revolution of FCNNs producing state-of-the-art performance on many medical image segmentation tasks. Various variants have been proposed, such as adding nested skip pathways in UNet++ (25), but the improvement in segmentation accuracy comes at the expense of more memory requirement and longer inference time. Replacing the standard encoder of U-Net with specialized convolutional neural network architectures for image classification has also been explored, such as using residual networks (ResNets) (26), squeeze-and-excitation networks (27), and aggregated residual transformations (28) in the U-Net encoder architecture.

### 2.2 Vision transformers

Transformers have lately gained popularity in computer vision applications. Dosovitskiy et al. (13) used large-scale pre-training and fine-tuning of a pure transformer to achieve state-of-the-art performance on image classification datasets. In particular, the authors developed Vision Transformer (ViT), a model that converts an input image into a sequence of patches and passes it to a transformer encoder and a multilayer perceptron to produce the desired output class.

Five key ideas largely contributed to the superior performance of ViT on vision tasks, most of which are borrowed from the original transformer architecture:

1. *Patch tokenization*: To mimic the sequential nature of the transformer’s text inputs, the input image is sliced up into square patches which are flattened into one-dimensional sequences before applying a linear projection to map each patch into a desired higher-dimensional embedding.
2. *Positional embeddings*: ViT uses learnable positional embeddings which are added to the projected patch embeddings before they are fed into the transformer encoder. Since each of the operations in the transformer encoder treats its inputs as a set (i.e., if the input embeddings are permuted, the outputs are also permuted, thus the order of patches is not important for the encoder), positional embeddings learn the important relative position of the patches with respect to the original input image.
3. *Multi-head attention*: The multi-head attention block in the transformer encoder allows input embeddings to communicate with each other so that they can share useful information. Compared to single-head attention, multi-head attention allows patches to send multiple messages to each other by performing multiple attention operations in parallel.
4. *MLP for local features*: Applying a two-layer multilayer perceptron (MLP) independently on each embedding allows the embeddings to focus on learning local information they each possess after they have communicated with each other through the multi-head attention block.
5. *Residual connections*: The use of residual connections help with optimization by avoiding vanishing gradients to help with gradient flow and by allowing the subunits in ViT to focus on learning the residual mapping than to optimize the original, unreferenced mapping.

However, despite the apparent success of ViTs, they have a couple of disadvantages over FCNNs. First, the self-attention mechanism in ViTs and other modern transformers has quadratic time- and space-complexity with respect to the size of the input. Keles et al. (29) has mathematically established quadratic lower bounds on the running time of self-attention. This quadratic barrier was proven to hold even if windowing, striding, or committing additive and multiplicative errors in the computation of self-attention were allowed. This quadratic runtime translates to slower processing of high-resolution or large inputs which may inadvertently increase the overall latency of ML systems that use transformers in their backend.

Another drawback of using ViTs is their requirement of large-scale pre-training in order to learn locality and translation equivariance. These two properties are desired model attributes for vision tasks on images with rigid structure such as chest radiographs and on images with repeated elements distributed across different locations. Unlike in ViTs where they still have to be trained on large datasets just to learn these two properties, FCNNs inherently have these two strong inductive biases. As studied in the original paper (13), ViT required over 303 million images for pre-training before it was able to beat the most superior CNN in their experiments. Thus, FCNNs are still preferred in lower-data regimes as not many researchers have access to very large labeled datasets and enough hardware to run similar experiments at scale. This is the case for medical image segmentation tasks where data is costly to collect, annotate, and verify.

In an effort to address these locality and translational equivariance issues, hierarchical vision transformers with various resolutions and spatial embeddings have been proposed recently (30–32). Borrowing the sliding window approach of FCNNs, hierarchical ViTs such as Swin Transformers (30) compute self-attention within a local window rather than globally. They employ patch merging to gradually lower the resolution of features in the transformer layers, similar to how the feature maps of a standard ConvNet increase in number but decrease in spatial dimension as one goes deeper in the network.

### 2.3 ConvNeXt models

In the previous section we have seen how models like hierarchical ViTs have proposed architectural changes to the original ViT to mimic some desirable behavior of FCNNs such as locality and translational equivariance. Recent work have tried the other direction of modernizing FCNNs to make them resemble transformers. In particular, ConvNeXt (33) is constructed entirely from standard ConvNet modules while adopting design choices from ViTs. The authors started with the standard ResNet-50 model and tweaked it by applying five techniques. First, they implemented some macro design changes such as following the stage compute ratio used in Swin Transformers and replacing the ResNet’s stem cell with a patchification layer to generate non-overlapping patches just like in ViTs. Second, the ConvNeXt authors used depthwise convolution used in ResNeXt (28) models to mix information solely in the spatial dimension which is comparable to the per-channel operation in ViT’s self-attention mechanism. Third, they implemented a similar inverted bottleneck design used in ViT’s transformer encoder where the hidden dimension of the MLP block is four times wider than the input dimension. Fourth, the authors increased the convolution kernel sizes from 3×3 to 7×7, copying the size of the sliding window in Swin Transformers. Fifth, they also implemented some micro design changes similar to those in ViTs: they applied fewer activation functions and normalization layers, replaced Rectified Linear Unit (ReLU) with Gaussian Error Linear Unit (34) (GELU), and substituted BatchNorm (35) with Layer Normalization (36) (LN). We note that ConvNeXt does not require specialized modules such as shifted window attention and relative position biases used in Swin Transformers.

Results from the original paper (33) show that the family of ConvNeXt models can compete favorably with ViT and its variants in terms of accuracy and scalability while being more efficient and much simpler in design. Similar to ConvNeXt, we explore the FCNN design space in this study to come up with a segmentation model constructed entirely from ConvNet modules but inspired by ViT techniques. We test our model’s limits on the dense prediction task of segmenting pneumothorax on chest radiographs.

### 2.4 Related studies on pneumothorax detection and segmentation

Deep learning has already been previously used to both detect and segment pneumothorax on chest radiographs. While models can be trained separately for each of the two tasks of classification and segmentation, models that can perform both tasks at the same time are preferred in the deployment setting as these models significantly reduce the memory footprint and inference time. Jakhar et al. in (37) used a conventional U-Net with pre-trained weights of a ResNet encoder backbone for segmenting pneumothoraces. In (38), the authors replaced the usual concatenation operations in the skip connection of U-Net with content-adaptive convolution (39), resulting to a 0.68% gain on the mean Dice similarity coefficient for pneumothorax segmentation. Hongyu et al. (40) employed a Mask R-CNN (41) using a ResNet-50 as a backbone feature pyramid network (FPN) (42) for detecting and segmenting pneumothorax. Whereas, in (43), Abedalla et al. used weighted averaging of four encoder-decoder networks based on U-Net which produced significant increases in classification and segmentation metrics but at the expense of larger memory footprint due to ensembling. Similar to (40), Malhotra et al. in (44) used a Mask R-CNN but with a ResNet-101 as its FPN for segmenting pneumothorax on chest X-rays. Our study proposes to tackle the dual task of pneumothorax detection and segmentation using a patch-based fully convolutional encoder-decoder network that aims to combine the advantages of FCNNs and ViTs while utilizing only convolutional modules to bypass the quadratic complexity of ViT’s self-attention mechanism.

## 3 Proposed architecture

**Figure 1:**
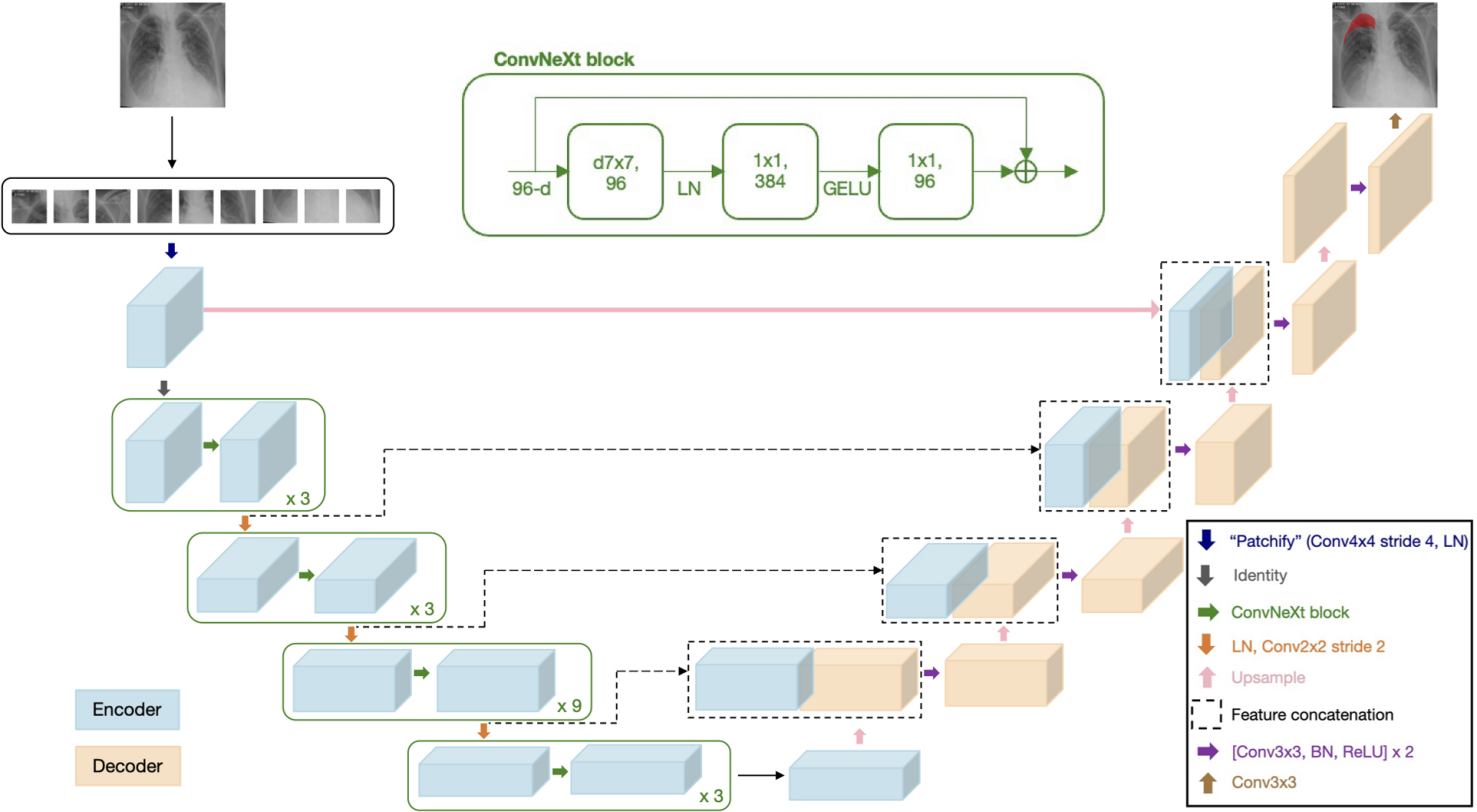
The encoder-decoder architecture of P-DeSeRay for pneumothorax detection and segmentation.

**Figure 2:**
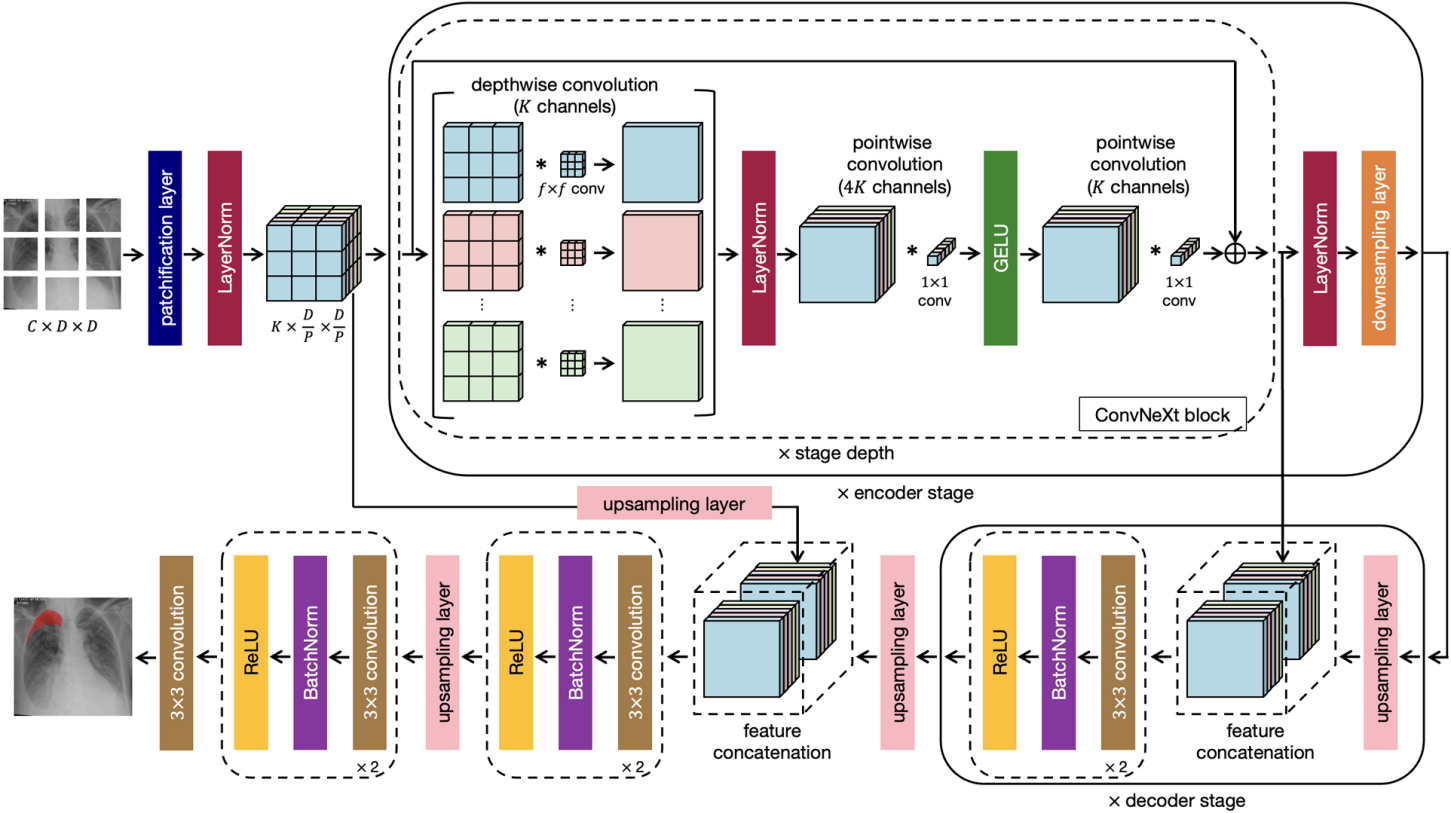
The same encoder-decoder architecture highlighting the operations used in the P-DeSeRay model.

This research work proposes a novel architecture that integrates the patch-based ConvNeXt encoder with the U-Net decoder, which we name P-DeSeRay, short for Pneumothorax Detection and Segmentation on Chest X-rays. Figure 1 visualizes the overall structure of the proposed model. Figure 2 highlights the operations used in P-DeSeRay and how our model differs from the seminal U-Net architecture. P-DeSeRay consists of a sequence of contracting ConvNeXt encoder blocks followed by a stack of expanding convolutional decoder blocks.

Adopting the transformers’ aggressive transformation of inputs to a 1D sequence of vector embeddings, we create a 1D sequence of a 2D input image $x\in R^{H\times W\times C}$ with resolution (H,W) and C input channels by applying a convolutional layer with a 4×4 kernel with stride 4. This non-overlapping convolution forms the patchification layer in the encoder’s stem. This layer partitions the input image into P×P patches (P=4) and projects them into a K-dimensional embedding space. We patterned our choice of K=96 to have the same number of channels as Swin-T architecture (30). We apply Layer Normalization (36) (LayerNorm or LN) right after the patch embedding layer for regularization.

After the encoder’s stem, we apply a stack of ConvNeXt blocks each comprising of 7×7 depthwise convolutions followed by pointwise (1×1) convolutions. We note that this combination separates spatial and channel mixing. On one hand, a depthwise convolution mixes information in the spatial dimension and operates on a per-channel basis similar to the weighted sum operation in the self-attention mechanism employed in transformers. On the other, a pointwise convolution mixes information in the channel dimension and operates on a per-pixel basis. This separation of mixing operations is a strategy exploited in ViTs. We visualize in Figure 2 how depthwise and pointwise convolutions perform spatial and channel mixing, respectively.

Two non-linearities are introduced between the depthwise and 1×1 convolutions. Mimicking one of the regularization techniques applied in transformers, LayerNorm is used after the 96 7×7 depthwise convolutions. Gaussian Error Linear Unit (34) (GELU) is employed as the activation function of the output from the 384 1×1 convolutions. Borrowing the identity mapping strategy introduced in the seminal ResNet, a residual connection is used between the input filters of the ConvNeXt block to the output of the last batch of 96 1×1 convolutions. A diagram of the ConvNeXt block is shown in Figure 1, and its operations are visualized in Figure 2. We note how the ConvNeXt block forms an inverted bottleneck design for the feature map, expanding the initial 96 channels to 384 (an expansion ratio of 4) before squeezing the features back to 96 channels. This inverted bottleneck design is widely used in Transformers and in advanced ConvNet architectures such as MobileNetV4 (45).

Similar to Swin-T, the ConvNeXt blocks are grouped into stages following a compute ratio of 1:1:3:1. In particular, the number of blocks in each of the 4 stages are 3, 3, 9, and 3 respectively. To adopt the hierarchical feature construction implemented in conventional CNNs and Swin Transformers, a 2×2 convolutional layer with stride 2 is used for spatial downsampling at the start of each encoder stage except the first one. A LN layer is applied before each downsampling to help stabilize training.

Akin to U-Net’s strategy of learning to assemble more precise outputs through the insertions of recovered spatial information at different resolutions from the encoder, the output of each encoder stage serves as an additional input to a decoder stage via skip connections. In addition, the encoder stem is also connected to a decoder stage. Because of the aggressive downsampling of our input image in the stem’s patchification layer, we needed to upsample the stem’s output feature map by a factor of 2 for it to match the dimensions of the feature map of its skip-connected decoder stage.

At the last encoder stage, we use a deconvolutional layer to its output feature map to resize its resolution by a factor of 2. Afterwards, we concatenate the upsampled feature map with the output of the previous encoder stage, and feed them to a decoder stage which consists of two decoder blocks each comprising a 3×3 convolutional layer followed by a BatchNorm (35) layer and a Rectified Linear Unit (46) (ReLU) activation. This procedure is repeated for all subsequent stages including the encoder stem’s output feature map. The output from the stem’s connected decoder stage is then upsampled by a factor of 2 and fed into a final decoder stage before applying a 3×3 convolutional layer in order to yield pixel-wise segmentation maps. Table 1 summarizes the architecture of P-DeSeRay. In the table, sc refers to the scaling of the output by a learnable gamma vector while res stands for the residual connection employed between the input of the encoder stage and its preliminary output.

**Table 1:** Detailed architecture specifications for P-DeSeRay.

Module/Layer	Operations	Output size
Image		(3,D,D)
Encoder stem (E-stem, “patchify” layer)	4×4,96, stride 4 LN	(96,D/4,D/4)
Encoder stage 0 (E-0)	$\left[\begin{array}{c} d7\times 7,96,\mathrm{LN}\\ 1\times 1,384,\mathrm{GELU},\mathrm{sc}\\ 1\times 1,96,\mathrm{res} \end{array}\right]\times 3$	(96,D/4,D/4)
Encoder stage 1 (E-1)	LN 2×2,192, stride 2	(192,D/8,D/8)
	$\left[\begin{array}{c} d7\times 7,192,\mathrm{LN}\\ 1\times 1,768,\mathrm{GELU},\mathrm{sc}\\ 1\times 1,192,\mathrm{res} \end{array}\right]\times 3$	
Encoder stage 2 (E-2)	LN 2×2,384, stride 2	(384,D/16,D/16)
	$\left[\begin{array}{c} d7\times 7,384,\mathrm{LN}\\ 1\times 1,1536,\mathrm{GELU},\mathrm{sc}\\ 1\times 1,384,\mathrm{res} \end{array}\right]\times 9$	
Encoder stage 3	LN	(768,D/32,D/32)
	2×2,768, stride 2	
	$\left[\begin{array}{c} d7\times 7,768,\mathrm{LN}\\ 1\times 1,3072,\mathrm{GELU},\mathrm{sc}\\ 1\times 1,768,\mathrm{res} \end{array}\right]\times 3$	
Decoder stage 0	Upsample	(256,D/16,D/16)
	Skip connection w/ E-2 out	
	$\left[\begin{array}{c} 3\times 3,256\\ \mathrm{BN},\mathrm{ReLU} \end{array}\right]\times 2$	
Decoder stage 1	Upsample	(128,D/8,D/8)
	Skip connection w/ E-1 out	
	$\left[\begin{array}{c} 3\times 3,128\\ \mathrm{BN},\mathrm{ReLU} \end{array}\right]\times 2$	
Decoder stage 2	Upsample	(64,D/4,D/4)
	Skip connection w/ E-0 out	
	$\left[\begin{array}{c} 3\times 3,64\\ \mathrm{BN},\mathrm{ReLU} \end{array}\right]\times 2$	
Decoder stage 3	Upsample	(32,D/2,D/2)
	Skip connection w/	
	upsampled E-stem out	
	$\left[\begin{array}{c} 3\times 3,32\\ \mathrm{BN},\mathrm{ReLU} \end{array}\right]\times 2$	
Decoder stage 4	Upsample	(16,D,D)
	$\left[\begin{array}{c} 3\times 3,16\\ \mathrm{BN},\mathrm{ReLU} \end{array}\right]\times 2$	
Segmentation head	3×3,1	(1,D,D)

## 4 Materials and methods

### 4.1 Data collection and annotation

For this study, chest X-ray images were collected from the Radiology Department of The Medical City (TMC) in Pasig City, Philippines. Three radiologists (two board certified radiologists and one radiology resident in training) collected X-rays in Digital Imaging and Communications in Medicine (DICOM) format retrospectively from patients who had their chest radiographs taken at the hospital from 2017 to 2022. The radiologists anonymized the DICOM files by removing personal identifiers such as the patient’s name, ID, birth date, sex, and age. The de-identified data from the hospital’s picture archiving and communication system (PACS) were then exported to a key-value DICOM database. We extracted important metadata from each DICOM file such as the accession number, the study date, the path to the DICOM file, and the projection used which is either posteroanterior (PA) or anteroposterior (AP). We stored these metadata in a relational database.

The radiographs were then meticulously annotated by our partner radiologists to generate their ground-truth masks. Prior to annotation, the images were extracted from the DICOM files using the Python package Pydicom (47). The images were then resized to a standard size of 2,048 × 2,048 using bicubic interpolation and the pixel values were normalized to a range of 0 to 255. We performed quality assurance on the labeled data to ensure that there are no duplicates and labeling errors.

**Figure 3:**
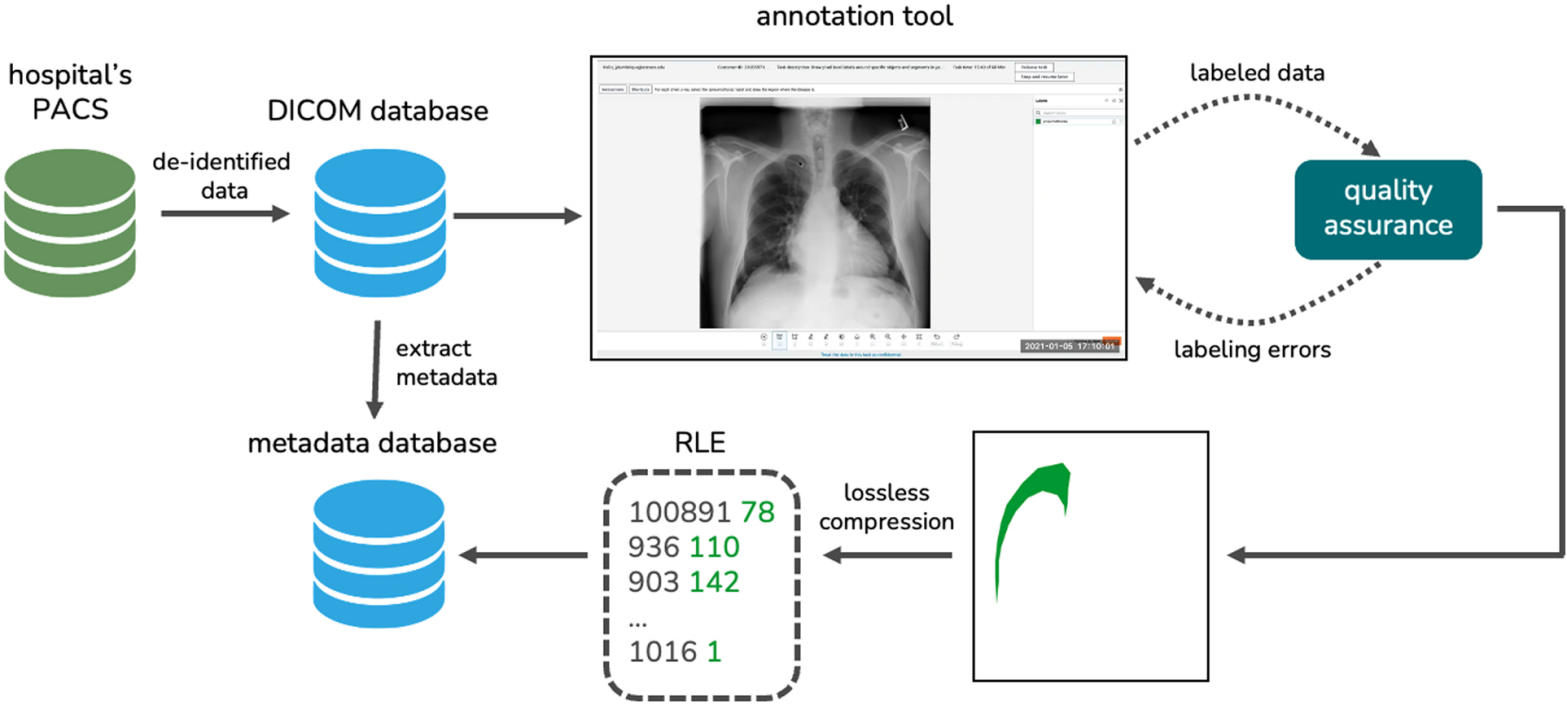
The data collection and annotation pipeline we implemented to generate our novel local dataset.

We developed our own radiograph annotation tool using Amazon SageMaker Ground Truth (48). Three radiologists used the in-house annotation tool to map out the ground-truth masks of the chest X-rays diagnosed with pneumothorax. These masks indicate the presence, location, and severity of pneumothorax on the dataset. For each radiograph, the consensus of the three radiologists’ annotations was used as the reference standard. The resulting ground-truth masks were reshaped to 1,024 × 1,024 images and were then converted to run-length encodings (RLEs) for efficient, lossless compression. These RLEs were added to our metadata database. Figure 3 visualizes our data collection and annotation pipeline.

### 4.2 Data splitting

For initial model training, we used the publicly available SIIM-ACR Pneumothorax Segmentation dataset (24), which contains 9,378 (77.8%) normal chest radiographs and 2,669 (22.2%) chest X-rays diagnosed with pneumothorax and their corresponding binary segmentation masks. For comparison with other works on the same dataset, the official train-test split imposed by SIIM-ACR was used. We further divided the SIIM train dataset into training and validation set using stratified cross validation which split the dataset into 5 folds with each fold having the same class distribution. P-DeSeRay’s initial parameters were derived using the SIIM training set. In order to prevent the issue of overfitting, the model was evaluated on the validation set to check whether the parameters were already optimized with regard to the loss function. In selecting which of the candidate models produces the highest segmentation and classification metrics, each model was tested on the separate SIIM test set which has not yet been seen by the model during its training phase.

The local TMC dataset was split into training and test sets using an 80%–20% ratio while preserving the class distribution across the two subsets. Transfer learning was conducted on the local training dataset. After fine-tuning, P-DeSeRay was evaluated on the local test set.

### 4.3 Data augmentation

Several data augmentation techniques were applied on the SIIM training and validation sets and on the TMC training dataset to make the segmentation model more robust. These include one of three exposure transformations (Contrast Limited Adaptive Histogram Equalization, Random Gamma Contrast, or Random Brightness Contrast), one of three blurs (Standard, Motion, or Median), Horizontal Flip, and affine transformations (translation, scaling, and rotation). All X-ray images were standardized into a 512×512 size and their RGB pixel values were normalized. The specific hyperparameters used for each data augmentation technique are summarized in Table 2.

**Table 2:** The hyperparameters used for the data augmentation techniques applied on the SIIM training and validation set and on the local training dataset.

Transformation class	Specific technique	Hyperparameters
Exposure (p=0.5)	Contrast Limited	Clip limit =4.0
	Adaptive Histogram	Tile grid size =(4,4)
	Equalization (CLAHE)	p=0.9
	Random Gamma	Gamma limit =(60,120)
	Contrast	p=0.9
	Random	Brightness limit =0.2
	Brightness	Contrast limit =0.2
	Contrast	p=0.9
Blur (p=0.5)	Standard Blur	Blur limit =4,p=1
	Motion Blur	Blur limit =4,p=1
	Median Blur	Blur limit =3,p=1
Flip (p=0.5)	Horizontal Flip	–
Affine (p=1)	Translation	Shift limit =0.2
	Scaling	Scale limit =0.2
	Rotation	Rotation limit =20

p indicates the probability that the specific transformation was applied.

### 4.4 Baseline fully convolutional networks

To compare the performance of P-DeSeRay with prior art, we constructed fully convolutional networks with U-Net as the base architecture. We sequentially introduced incremental architectural changes to the encoder in three stages:

1. ResNet-101 Encoder: We first replaced the conventional U-Net encoder with ResNet-101 (26), a deep convolutional neural network architecture implementing residual learning. The network comprises 101 layers organized into multiple residual blocks, each employing identity mappings with skip connections. The identity mappings allow gradients to flow directly through the network, effectively mitigating the vanishing gradient problem and enabling training of networks with unprecedented depth.
2. Squeeze-and-Excitation ResNet-101 (SE-ResNet-101) Encoder: We further enhanced the ResNet-101 encoder by incorporating squeeze-and-excitation (SE) blocks (27). SE blocks adaptively recalibrate channel-wise feature responses by explicitly modeling interdependencies between channels. The SE mechanism works in two steps: (i) squeeze operation, which aggregates feature maps globally to capture channel-wise global context, and (ii) excitation operation, which learns channel-wise attention weights. This allows the network to dynamically adjust feature representation importance.
3. Squeeze-and-Excitation ResNeXt-101 (SE-ResNeXt-101) Encoder: As a final architectural modification, we integrated aggregated residual transformations (ResNeXt) (28) with SE blocks. The ResNeXt architecture introduces the concept of cardinality, where multiple parallel transformation paths are aggregated within a block. In our implementation, we used internal dimension d=4 and cardinality C=32, which creates multiple grouped convolutions that capture diverse feature representations. By combining ResNeXt’s multi-path aggregation with SE blocks’ channel-wise attention, we created a more expressive and adaptive encoder.

The initial weights of the three encoders were pre-trained on ImageNet (49) and obtained from the Segmentation Models PyTorch package (50). All our models were trained using the same training process presented in the following section.

### 4.5 Training procedure

Each model was trained with a linear combination of Tversky and Focal Losses (51, 52). The Tversky loss $L_{\text{Tversky}}$ is calculated using the Tversky similarity index T, which is a generalization of the Dice similarity coefficient (DSC) that allows for flexibility in balancing false positives and false negatives. The Tversky loss aggregates across all N pixels in the image and is given by

$$L_{\;Tversky}(\beta )=1-T(\beta )$$

where

$$T(\beta )=\frac{\sum_{i=1}^{N}p_{0i}g_{0i}}{\sum_{i=1}^{N}p_{0i}g_{0i}+\beta \sum_{i=1}^{N}p_{0i}g_{1i}+(1-\beta )\sum_{i=1}^{N}p_{1i}g_{0i}},$$

such that in the model’s output, $p_{0i}$ is the probability that pixel i has pneumothorax and $p_{1i}=1-p_{0i}$ is the probability that pixel i does not have pneumothorax. Also, the ground truth label $g_{0i}$ is 1 if pixel i has pneumothorax and is 0 if pixel i does not have pneumothorax, and vice versa for $g_{1i}$. The Tversky index incorporates a penalty hyperparameter β∈[0,1] that penalizes false positives more than false negatives with higher values. The Tversky index simplifies to DSC when β=0.5.

The focal loss, on the other hand, is a variant of the widely used binary cross-entropy (CE) loss. Tuning a focusing hyperparameter γ≥0 in the focal loss allows the model to prioritize learning from difficult, misclassified samples over simple ones. As the value of γ is increased, the down-weighing of the loss contributions of easy, well-classified examples strengthens. When γ=0, the focal loss reduces to the binary CE loss. For the focal loss $L_{Focal}$ applied to our semantic segmentation task, we calculate the per-pixel focal loss and get the mean across all the pixels:

$$L_{\;Focal}(\gamma )=\frac{1}{N}\sum_{i=1}^{N}\left[-(1-p_{i,t}{)}^{\gamma }\log(p_{i,t})\right],$$

where

$$p_{i,t}=\left\{ \begin{array}{cc} p_{0i}\; & \mathrm{if}\;\mathrm{pixel}\;i\;\mathrm{has}\;\mathrm{pneumothorax}\;(g_{0i}=1)\;\\ p_{1i}\; & \mathrm{if}\;\mathrm{pixel}\;i\;\mathrm{does}\;\mathrm{not}\;\mathrm{have}\;\mathrm{pneumothorax}\;(g_{0i}=0)\; \end{array}\right..$$

Finally, the mixed loss $L_{mixed}$ we used for training our models is the sum of the Tversky and focal losses:

$$L_{\;mixed}=L_{\;Focal}+L_{\;Tversky}.$$

We evaluated the impact of different loss functions on model performance in our experiments. Specifically, we compared the mixed loss $L_{mixed}$ to the Tversky loss $L_{Tversky}$. We employed β=0.5 for both losses and used the optimal value γ=2 (52) for the focal loss component of the mixed loss. Adam (53) was used as the optimizer in all of the models, with an initial learning rate of 0.0005 that is progressively decreased until the loss function reaches a plateau. P-DeSeRay and the modified U-Net-based models were trained with a batch size of 8, while the conventional U-Net model was trained with a batch size of 16. The training data was shuffled for each epoch and early stopping was imposed by selecting the model checkpoint that produced the smallest validation loss.

A sigmoid function was applied pixelwise on the output segmentation mask from the model. The resulting probability map was turned into a binary mask through thresholding at p=0.5. For the detection task, pneumothorax is deemed to be present on the input chest X-ray if the output 512×512 binary mask has at least 3,500 (1.34%) activated pixels. Otherwise, the X-ray is predicted to not have pneumothorax. We used the open-source platform MLflow (54) to track our experiments and the various versions of our models and hyperparameters. Pertinent details on our training procedure are summarized in Table 3.

**Table 3:** Training procedure details for P-DeSeRay and the baseline models.

Parameter	Value
optimizer	Adam
learning rate	0.0005
	gradually reduced when the loss
	function has plateaued
batch size	8 or 16
epochs	50
	training data are shuffled every epoch,
	early stopping: picked the model
	checkpoint with lowest validation loss
threshold for binary mask	0.5
threshold for pneumothorax detection	≥3,500 activated pixels
	(≥1.34% of the 512 × 512 mask)

### 4.6 Evaluation metrics

To assess the performance of our model on the test data sets, the mean Dice similarity coefficient (DSC) (51) and the mean Intersection over Union (IoU) were used as segmentation metrics while the sensitivity, specificity, F1, and F2 scores were calculated to quantify the binary classification performance. The specific formulas for these metrics are as follows:

1. Dice similarity coefficient
  $$DSC=\frac{2\cdot |X\cap Y|}{\left|X|+|Y\right|},$$
  where X and Y are the sets representing the predicted and actual binary masks of a chest radiograph respectively, and the operation |⋅| indicates the cardinality of a set (i.e., the number of nonzero pixel-wise labels in a binary mask).
2. Intersection over Union (Jaccard index)
  $$IoU=\frac{\left|X\cap Y\right|}{\left|X|+|Y|-|X\cap Y\right|},$$
  where X and Y are defined similarly as in the previous DSC metric.
3. sensitivity (true positive rate, recall)
  $$Sensitivity=\frac{TP}{TP+FN},$$
  where TP is the number of true positives (on an image level) and FN indicates the number of false negatives.
4. specificity (true negative rate)
  $$Specificity=\frac{TN}{FP+TN},$$
  where TN is the number of true negatives and FP indicates the number of false positives.
5. F1 score
  $$F1\;score=2\cdot \frac{Precision\cdot Sensitivity}{Precision+Sensitivity},$$
  where precision is calculated as
  $$Precision=\frac{TP}{TP+FP}.$$
6. F2 score
  $$F2\;score=5\cdot \frac{Precision\cdot Sensitivity}{(4\cdot Precision)+Sensitivity}$$
  Similar to F1 score, the F2 score combines precision and sensitivity into one metric but it puts more weight on sensitivity than precision.

## 5 Results and discussion

### 5.1 Data collection and annotation results

Employing our multi-stage data collection approach, we constructed a novel dataset comprised of 1,039 de-identified chest radiographs obtained from patients admitted to TMC during the period 2017–2022. Out of these, 229 chest X-rays were diagnosed with pneumothorax as verified by their respective clinical reports. The other 810 radiographs were diagnosed as normal. In this section, we analyze the radiographs in the SIIM and TMC datasets in terms of (i) the laterality of pneumothorax, (ii) the size of the affected area, (iii) the radiograph’s projection.

**Figure 4:**
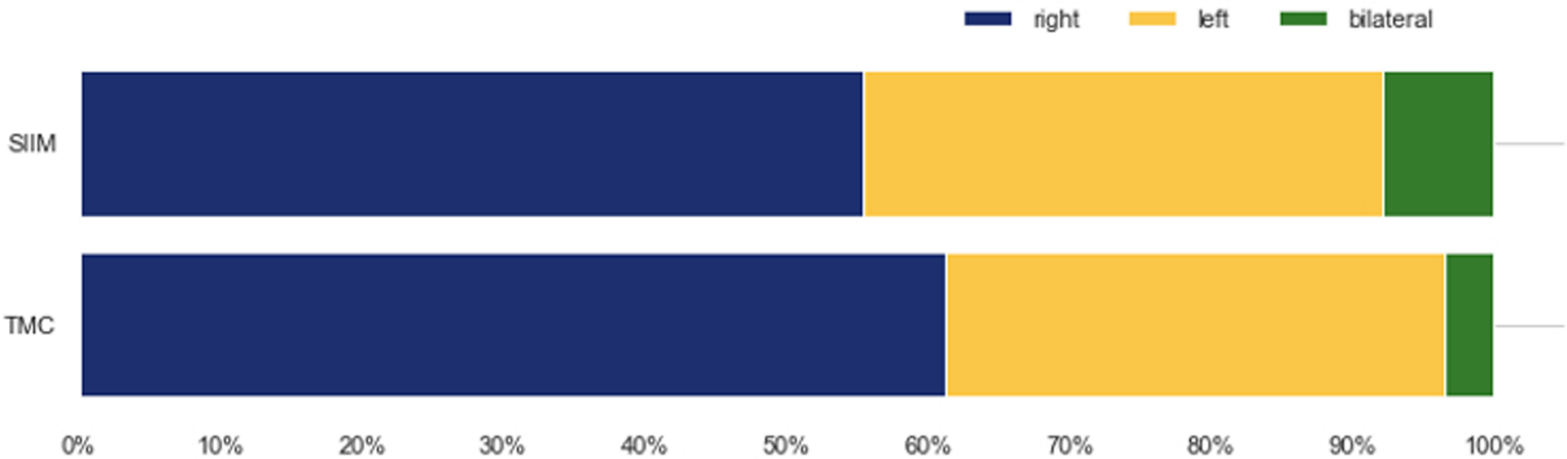
Laterality of pneumothorax on the affected chest X-rays in the SIIM and TMC datasets.

Our analysis of the TMC dataset revealed a laterality distribution of pneumothorax similar to the SIIM dataset (Figure 4). In the TMC data, 61.1% of detected pneumothoraces affected the right lung only, compared to 35.4% affecting the left lung only and 3.5% being bilateral. The SIIM dataset exhibited a comparable distribution, with 55.3% of pneumothoraces affecting the right lung only, 36.8% the left lung only, and 7.8% bilateral.

**Figure 5:**
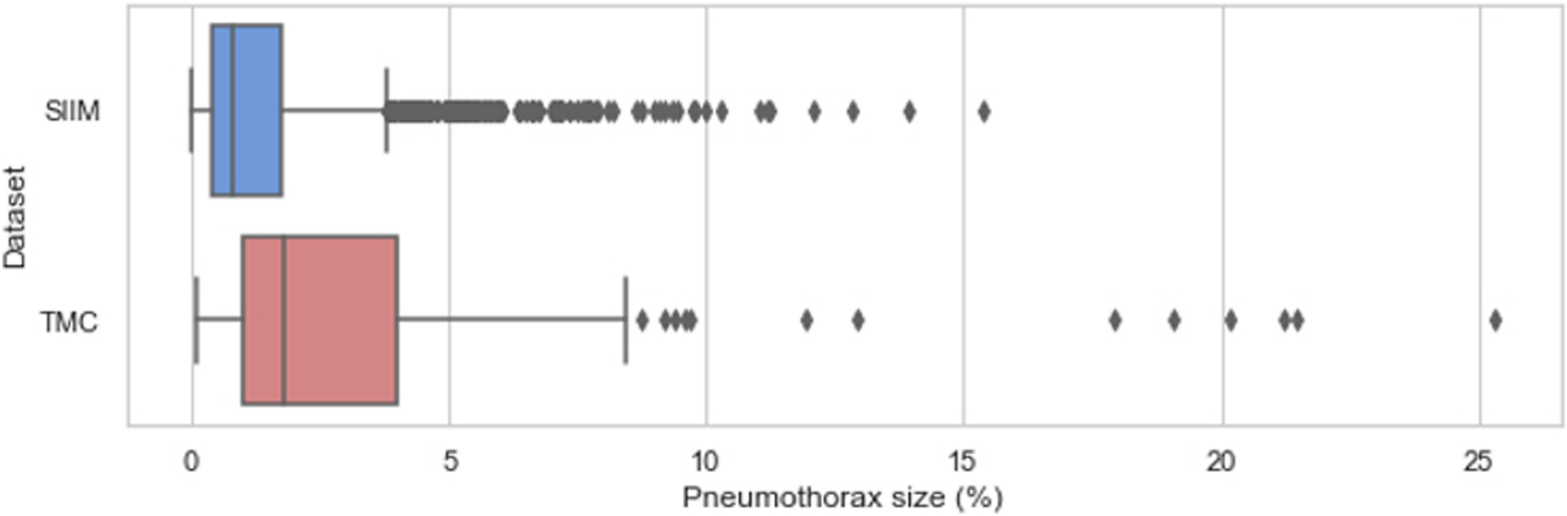
Sizes of pneumothorax (% of total image pixels) on the affected chest X-rays in the SIIM and TMC datasets.

On the other hand, the distribution of pneumothorax sizes in the SIIM and TMC datasets is visualized in Figure 5. Pneumothorax size was defined as the ratio of the ground-truth mask area to the total image area (i.e., the number of pixels with pneumothorax divided by the total image pixels). The TMC dataset exhibited statistically larger pneumothoraces (μ=3.23,σ=3.84) compared to the SIIM dataset (μ=1.37,σ=1.57).

**Figure 6:**
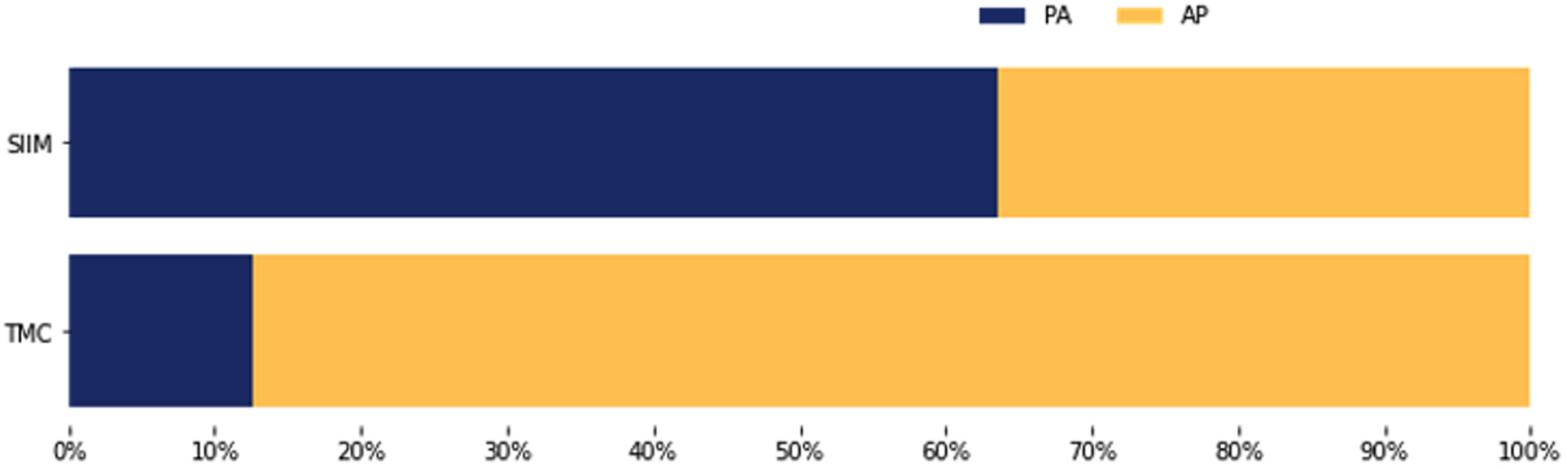
Projection of the chest X-rays diagnosed with pneumothorax in the SIIM and TMC datasets.

Moreover, the TMC dataset exhibited a distinct projection distribution compared to the SIIM dataset (Figure 6). In the TMC data, 87.3% of chest radiographs with pneumothorax were acquired using an anteroposterior (AP) view, while only 12.7% were captured in the posteroanterior (PA) projection. Conversely, the SIIM dataset showed a predominance of PA views (63.6%) for pneumothorax cases, with 36.4% acquired using the AP view.

### 5.2 Results on the SIIM test dataset

Table 4 summarizes the segmentation and detection performance of various models on the SIIM test dataset. As shown, P-DeSeRay achieved state-of-the-art segmentation performance with mean Dice Similarity Coefficient (mDSC) and mean Intersection over Union (mIoU) of 85.8% and 83.7%, respectively. This surpassed prior art models (37, 38, 40, 43, 44) and baseline U-Net models trained with Tversky loss, even with the implemented encoder modifications. We investigated the impact of these modifications on the U-Net architecture. A conventional U-Net model achieved an mDSC of 79.5% and an mIoU of 77.6%. Replacing the U-Net encoder with a ResNet-101 encoder improved the mDSC and mIoU to 81.6% and 80.5%, respectively (a gain of 2.1% and 2.9%). Further incorporating a squeeze-and-excitation block in each residual block of the ResNet-101 encoder resulted in a marginal increase of 0.3% for both mDSC and mIoU. Finally, adding aggregated residual transformations with an internal dimension of d=4 and cardinality C=32 led to a more substantial improvement of 1.3% and 1.2% in mDSC and mIoU, respectively. However, despite these architectural enhancements that boosted the baseline U-Net’s segmentation performance by 3.7% and 4.4% in mDSC and mIoU, P-DeSeRay trained with the Tversky loss function still outperformed the most complex modification (U-Net with SE-ResNeXt-101 encoder) by a significant margin of 2.6% and 1.7% in mDSC and mIoU, respectively.

**Table 4:** The models’ number of trainable parameters, segmentation and detection performance metrics (in %) on SIIM test dataset.

Models	#Params	mDSC	mIoU	F1	F2	Sensitivity	Specificity
Baseline: U-Net (original)	7.8M	79.5	77.6	67.1	66.4	65.9	91.9
+ ResNet101 encoder	51.5M	81.6	80.5	66.9	64.8	63.4	93.0
+ squeeze-and-excitation blocks	56.3M	81.9	80.8	67.9	68.5	69.0	90.9
+ aggregated residual	55.9M	83.2	82.0	73.5	67.8	64.5	97.0
transformations							
(d=4,C=32)							
+ focal loss (γ=2)	55.9M	85.6	83.7	73.8	68.8	65.9	96.6
P-DeSeRay (Ours)	31.9M	85.8	83.7	76.5	70.4	66.9	**97.9**
+ focal loss (γ=2)	31.9M	**86.7**	**84.7**	**77.1**	**73.6**	71.4	96.3
Mostayed et al., 2019 (38)	7.1M	76.0	–	–	–	–	–
Hongyu et al., 2020 (40)	–	82.0	81.0	60.0	–	**78.0**	78.0
Abdella et al., 2021^∗^ (43)	25.6M	–	80.3	63.2	–	56.9	–
Jakhar et al., 2019^∗^ (37)	–	84.3	82.6	–	–	–	–
Malhotra et al., 2022 (44)	–	–	82.9	–	–	–	–

Bold values highlight the highest performance score in each column.

^∗^Indicates that the reference work tested on a subset of the SIIM train set rather than on the official SIIM test set.

P-DeSeRay also achieved state-of-the-art performance in binary classification metrics, surpassing all models trained with the Tversky loss function and previously published models. P-DeSeRay trained solely with Tversky loss achieved an F1 score of 76.5%, an F2 score of 70.4%, sensitivity of 66.9%, and specificity of 97.9%. The conventional U-Net model served as a baseline, achieving F1, F2, sensitivity, and specificity scores of 67.1%, 66.4%, 65.9%, and 91.9%, respectively. Replacing the U-Net encoder with a ResNet-101 encoder improved specificity by 1.1% but came at the expense of F1, F2, and sensitivity scores, which decreased slightly. Further architectural modifications yielded mixed results. Incorporating squeeze-and-excitation blocks improved F1, F2, and sensitivity scores but decreased specificity. Adding aggregated residual transformations led to substantial gains in F1 score and specificity but decreased F2 score and sensitivity. Overall, these changes on the U-Net resulted in net improvements in F1, F2, and specificity but a slight decrease in sensitivity. Importantly, despite these enhancements to the U-Net architecture, the patch-based P-DeSeRay model still outperformed these baseline models in pneumothorax detection across all classification metrics. P-DeSeRay trained with Tversky loss significantly surpassed the most complex U-Net modification (with SE-ResNeXt-101 encoder) by a margin of 3.0%, 2.6%, 2.4%, and 0.9% in F1, F2, sensitivity, and specificity scores, respectively. Notably, P-DeSeRay also outperformed the F1, sensitivity, and specificity scores reported in previous studies (40, 43).

Our ablation study (Table 4) examined the effect of different loss functions. Training models with an unweighted mixed loss combining Tversky and Focal losses improved both segmentation and detection performance compared to Tversky loss alone. P-DeSeRay trained with the mixed loss achieved superior segmentation performance, with gains of 0.9% and 1.0% in mDSC and mIoU, respectively. Notably, P-DeSeRay also exhibited improved classification with the mixed loss, achieving increases of 0.6% and 3.2% in F1 and F2 scores. However, as expected with these metrics, the mixed loss increased P-DeSeRay’s sensitivity by 4.5% but decreased its specificity by 1.6%, reflecting the known trade-off between the two (55).

The positive impact of the mixed loss and the sensitivity-specificity trade-off observed with P-DeSeRay were also evident in the baseline U-Net models (refer to Table 4). Notably, employing the mixed loss alongside architectural changes significantly improved the U-Net’s mDSC and mIoU by 2.4% and 1.7%, respectively, with minimal reductions in specificity (0.4%). However, P-DeSeRay trained solely with Tversky loss still surpassed the performance of these U-Net models even when they leveraged the mixed loss. This finding underscores the significant contribution of our proposed patch-based fully convolutional encoder-decoder architecture to pneumothorax segmentation and detection.

P-DeSeRay offers not only superior performance but also improved computational efficiency. While achieving state-of-the-art results, P-DeSeRay is a medium-sized network with only 31.9 million parameters, significantly less than the modified baseline U-Net models, each exceeding 51.5 million parameters. This translates to a reduction in computational complexity of over 38%. P-DeSeRay’s efficiency advantage persists even when compared to similar-sized models from prior art, such as the one presented in (43). In conclusion, our P-DeSeRay model, trained with an unweighted combination of Tversky and focal losses, delivers superior segmentation and classification performance on the SIIM dataset while maintaining computational efficiency.

### 5.3 Results on the TMC test dataset

Table 5 summarizes P-DeSeRay’s performance after fine-tuning on the TMC training data. P-DeSeRay achieved state-of-the-art segmentation performance on the TMC test set, with a mDSC of 90.9% and a mIoU of 88.6%. P-DeSeRay also demonstrated exceptional pneumothorax detection capabilities, achieving a specificity of 98.6%, precision of 94.6%, and accuracy of 95.4%. Notably, the F1 and F2 scores for detection were 88.9% and 85.8%, respectively. Furthermore, P-DeSeRay’s sensitivity of 83.8% significantly surpasses the reported pooled sensitivity of radiologists (45.7%) for pneumothorax detection on X-rays (56). The model efficiently processed radiographs from the local test set with an average inference time of 0.3184 s per image.

**Table 5:** Segmentation and classification performance (in %) of P-DeSeRay on the TMC test dataset.

Metric	Radiologists’ level (56)	P-DeSeRay
mDSC	–	90.9
mIoU	–	88.6
Sensitivity	45.7	83.8
Specificity	99.6	98.6
F1	–	88.9
F2	–	85.8
Precision	–	94.6
Accuracy	–	95.4

**Figure 7:**
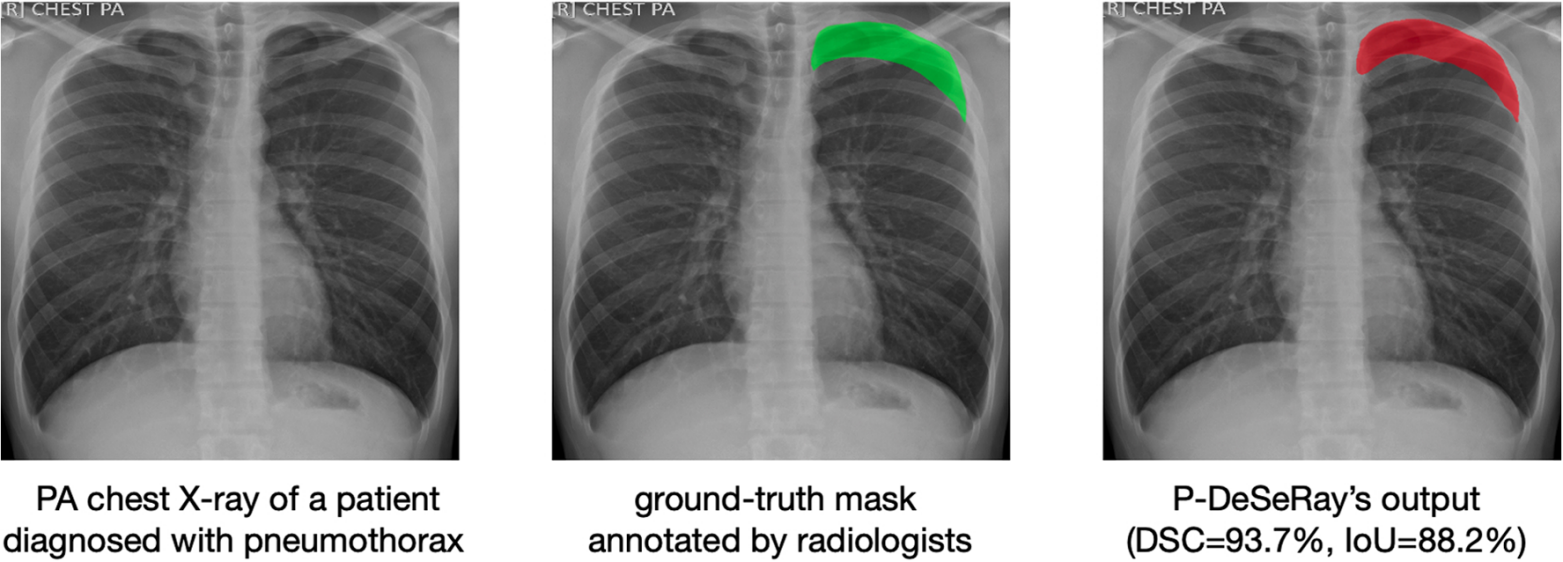
A representative case in the TMC test set with its corresponding ground-truth binary mask (in green color) as assessed by radiologists and P-DeSeRay’s predicted output mask (in red color) with a DSC of 93.7% and IoU of 88.2%. PA, posteroanterior.

A representative case from the TMC dataset is visualized in Figure 7, showcasing a sample de-identified chest X-ray, its ground-truth mask, and the predicted output mask generated by our model. When compared to the ground-truth mask, P-DeSeRay’s output is smoother in outlining the affected area around the lung apex.

### 5.4 Understanding P-DeSeRay’s superior performance: a look at key architectural components

P-DeSeRay’s superior performance in pneumothorax segmentation and detection can be attributed to several key architectural components that draw inspiration from Vision Transformers while maintaining the efficiency of fully convolutional neural networks: (1) the use of patchification layer, (2) the separation of spatial and channel mixing, (3) the inverted bottleneck design in our encoder blocks and (4) the use of the patch embeddings as decoder inputs.

#### 5.4.1 P-DeSeRay’s patchification layer and embeddings

Unlike standard FCNNs that process the entire image at once, P-DeSeRay introduces a unique change in input representation through its patchification layer. This layer strategically divides the input image into smaller, non-overlapping patches. These patches are then linearly embedded into a higher dimensional space, allowing the network to capture richer localized features crucial for pneumothorax detection. Finally, the resulting patch embeddings are directly fed into our encoder blocks for further processing. This approach shares similarities with the successful use of tokenization and embedding in Transformer architectures. Like Transformers and ViTs that tokenize their inputs before feeding them to self-attention layers, P-DeSeRay leverages patchification and embedding to prepare the input for efficient processing by the subsequent encoder blocks.

#### 5.4.2 Separation of spatial and channel mixing

P-DeSeRay employs a distinct approach compared to standard FCNNs in how it mixes information within the network. Unlike FCNNs that rely on traditional 2D convolutions for both spatial (across pixels) and channel-wise (across feature maps) mixing, P-DeSeRay separates these operations. This separation strategy is implemented in ViT through its self-attention operation which performs the spatial mixing, and the succeeding MLP which executes the channel mixing. P-DeSeRay implements a similar separation strategy through depthwise separable convolutions to avoid the quadratic complexity inherent in ViT’s self-attention mechanism. As visualized in Figure 2, these convolutions consist of two sequential steps:

• Depthwise convolution: This step focuses on spatial mixing, applying filters to each channel independently, preserving spatial information.
• Pointwise convolution (1x1 convolution): This step performs channel-wise mixing, combining information across channels while maintaining the spatial resolution obtained in the depthwise step.

This separation of spatial and channel mixing allows P-DeSeRay to potentially learn more intricate relationships between features in the image. By focusing on spatial information first, the network might be better equipped to capture subtle spatial patterns indicative of pneumothorax, ultimately leading to improved segmentation and detection performance.

#### 5.4.3 Inverted bottleneck design in encoder blocks

P-DeSeRay utilizes an inverted bottleneck design within its encoder blocks. This design differs from the standard bottleneck design used in ResNet, SENet, and ResNeXt blocks we employed in our modified U-Net models. Here’s a breakdown of the key differences:

1. Standard Bottleneck: In a standard bottleneck, the input features are first compressed to a lower dimension and then expanded back to a higher dimension.
2. Inverted Bottleneck: P-DeSeRay’s inverted bottleneck design takes the opposite approach. The input features are initially expanded to a higher dimension using an expansion ratio of 4 in this case. This expansion allows the model to extract richer channel-dependent information. Subsequently, the features are projected back to a lower dimension, effectively aggregating the channel-wise dependencies and retaining the most important information.

We note that ViTs and other advanced ConvNet architectures such as MobileNetV4 also use an inverted bottleneck. In particular, Vision Transformers implements it in their MLP layers, expanding the channel dimension by a factor of 4 before projecting back. The inverted bottleneck design offers several benefits to our P-DeSeRay model. First, the initial expansion facilitates the extraction of more intricate channel-dependent features within the intermediate higher-dimensional space. Second, the subsequent channel reduction effectively aggregates this information, ensuring that the most critical details are retained. Third, the inverted bottleneck design allows P-DeSeRay to extract complex features from patches while maintaining computational efficiency. This is particularly advantageous for medical image analysis tasks like pneumothorax segmentation, where preserving detail is crucial for accurate diagnosis.

#### 5.4.4 Patch embeddings as decoder inputs

After processing by the encoder blocks, P-DeSeRay leverages the patch embeddings as inputs to the decoder block. To accommodate this, P-DeSeRay employs an upsampling step to increase the resolution of the patch embeddings by a factor of 2 before concatenation with the feature map from the corresponding decoder stage (see Figure 1). The patchification layer at the beginning of P-DeSeRay’s architecture performs a significant downsampling step. This initial downsampling serves two key purposes:

• Increased Receptive Field: It increases the effective receptive field size, allowing the network to capture long-range dependencies in the image. This is crucial for efficiently performing spatial mixing on distant pixel locations.
• Information Retention: Despite the downsampling, P-DeSeRay’s patchification process retains substantial information from the X-ray image due to the rich content within the patch embeddings.

By concatenating the upsampled patch embeddings with the decoder block’s feature maps, P-DeSeRay can reconstruct the spatial relationships between the processed image patches. This is particularly beneficial for pneumothorax segmentation, where preserving the spatial context of features is critical for accurate delineation of the collapsed lung region.

Overall, P-DeSeRay’s distinct architectural components work synergistically to achieve superior performance in pneumothorax segmentation and detection. The patchification layer, separation of spatial and channel mixing, inverted bottleneck design, and use of patch embeddings as decoder inputs all contribute to the network’s ability to extract meaningful features and reconstruct an accurate segmentation map.

## 6 Conclusion and recommendations

This study investigated the development and evaluation of a deep learning model for automatic pneumothorax segmentation and detection in chest radiographs. Pneumothorax is a life-threatening condition that can affect individuals of any age and health background, often with a significant recurrence rate. Early and accurate detection is crucial for optimal patient outcomes. However, diagnosing pneumothorax solely based on chest X-rays can be challenging due to the subtle visual cues, such as a thin displaced pleural line. Furthermore, determining the severity and location of pneumothorax on the X-ray is critical for guiding treatment decisions. By automating these tasks, our research has the potential to improve diagnostic accuracy and efficiency, particularly in resource-limited settings with radiologist shortages.

In this work, we propose P-DeSeRay, a patch-based fully convolutional encoder-decoder network composed of a convolutional encoder that directly utilizes the embedded 2D patches and a skip-connected decoder that combines extracted representations at different resolutions from the encoder. P-DeSeRay surpassed the state-of-the-art in pneumothorax segmentation and detection on both a public dataset and an independent dataset we curated from The Medical City’s Radiology Department. Our model outperformed not only other ConvNets but also prior research and even the reported sensitivity of radiologists on chest X-rays.

Demonstrating its suitability for real-world deployment, P-DeSeRay is computationally efficient. It boasts a reduction in complexity of over 38% compared to modified U-Net models and requires only 0.3184 s for average inference per image. This study demonstrates the effectiveness and efficiency of modern ConvNets like P-DeSeRay for medical image segmentation and classification tasks. Unlike ViTs, P-DeSeRay relies solely on standard convolutional modules, avoiding the quadratic complexity challenges associated with ViT’s self-attention mechanism.

In order to make our study cost-efficient in a data-scarce setting, we applied transfer learning from a public dataset to a smaller locally curated dataset. Moreover, ablation studies show that training P-DeSeRay using an unweighted linear combination of Tversky and Focal losses has significantly increased the segmentation and detection performance when compared to using Tversky loss solely. These training strategies, coupled with the proposed novel architecture, have shown significant contributions to P-DeSeRay’s performance.

This study hopes to contribute to the growing evidence of the effectiveness of deep learning models in performing modern medical imaging and radiological tasks. P-DeSeRay demonstrated fast and reliable detection and segmentation of pneumothorax on chest radiographs. When deployed to the clinical setting, our model has the potential to significantly increase the radiologists’ diagnostic performance and reduce backlogs by optimizing the reading time per radiograph. Furthermore, P-DeSeRay has the potential to reduce diagnostic errors in pneumothorax detection for radiology residents by providing a second opinion and highlighting subtle pneumothoraces that might be missed on visual inspection.

For future work, we aim to integrate P-DeSeRay into a computer-aided diagnosis software prototype. This would serve as a valuable clinical decision-support tool for radiologists and radiology residents, especially those located in developing countries with resource limitations. Conducting multi-center studies with diverse patient populations would also be beneficial in generalizing our findings and in paving the way for wider clinical adoption.

## Data Availability

The datasets presented in this article are not readily available because of data security and confidentiality. Requests to access the datasets should be directed to jakovivan.dumbrique@gmail.com.
